# ASIC2 Subunits Facilitate Expression at the Cell Surface and Confer Regulation by PSD-95

**DOI:** 10.1371/journal.pone.0093797

**Published:** 2014-04-03

**Authors:** Anne Marie S. Harding, Nobuyoshi Kusama, Tomonori Hattori, Mamta Gautam, Christopher J. Benson

**Affiliations:** 1 Department of Internal Medicine, Roy J. and Lucille A. Carver College of Medicine, University of Iowa, Iowa City, Iowa, United States of America; 2 Department of Veterans Medical Center, Iowa City, Iowa, United States of America; University of Pittsburgh, School of Medicine, United States of America

## Abstract

Acid-sensing ion channels (ASICs) are Na^+^ channels activated by changes in pH within the peripheral and central nervous systems. Several different isoforms of ASICs combine to form trimeric channels, and their properties are determined by their subunit composition. ASIC2 subunits are widely expressed throughout the brain, where they heteromultimerize with their partnering subunit, ASIC1a. However, ASIC2 contributes little to the pH sensitivity of the channels, and so its function is not well understood. We found that ASIC2 increased cell surface levels of the channel when it is coexpressed with ASIC1a, and genetic deletion of *ASIC2* reduced acid-evoked current amplitude in mouse hippocampal neurons. Additionally, ASIC2a interacted with the neuronal synaptic scaffolding protein PSD-95, and PSD-95 reduced cell surface expression and current amplitude in ASICs that contain ASIC2a. Overexpression of PSD-95 also reduced acid-evoked current amplitude in hippocampal neurons. This result was dependent upon ASIC2 since the effect of PSD-95 was abolished in *ASIC2−/−* neurons. These results lend support to an emerging role of ASIC2 in the targeting of ASICs to surface membranes, and allows for interaction with PSD-95 to regulate these processes.

## Introduction

ASICs are H^+^-gated Na^+^-conducting ion channels predominantly expressed in the nervous systems of chordates. In mammals, 4 genes (*ASIC1-4*) encode for at least 6 different ASIC subunits [1]. In rodents, ASIC1 and ASIC2 have alternative splice forms (ASIC1a and -1b; -2a and -2b, respectively), which differ in the intracellular N-termini and first transmembrane domains. ASICs are assembled as either homo- or hetero-trimeric complexes, and each combination of subunits forms channels with unique biophysical properties [2], [3], [4]. ASIC1a is the major determinant of pH-activated currents in central nervous system (CNS) neurons [5], [6], [7]. Expressed at the soma and in dendrites, including post-synaptic sites, ASIC1a is positioned to sense local pH changes within the brain [6], [8]. ASIC1a plays a role in some forms of learning and memory, and it seems particularly important for innate fear responses and acquired fear-conditioned behaviors [6], [9], [10], [11]. In addition, pharmacological or genetic perturbation of ASIC1 alters outcomes in mouse models of stroke and seizures – pathological conditions associated with tissue acidosis [12], [13]. In contrast to ASIC1a, ASIC3 is predominantly expressed in peripheral sensory neurons, where it contributes to pain sensation, including that from the heart and skeletal muscle [14], [15], [16], [17], [18]. Similarly, ASIC1b is primarily expressed in the periphery and participates in pain [19], [20]. ASIC4 does not contribute to H^+^-gated currents, and its function in mammals is largely unknown [21], [22].

ASIC2 subunits are widely expressed in both the central and peripheral nervous systems [23], [24], [25]. However the function of ASIC2 is less well understood. The splice variant ASIC2a forms H^+^-gated homomeric channels when expressed alone, but ASIC2a homomers are far less pH sensitive than other ASICs – requiring pH levels below 5.0 to be activated. Moreover, ASIC2a homomeric-like currents have not been described in wildtype native neurons. ASIC2b does not form functionally active channels when expressed alone. On the other hand, ASIC2 subunits can heteromultimerize with other subunits to generate channels with unique functional properties [3], [4], [26], [27]. For example, we recently found that ASIC2 and -3 function as heteromultimeric channels (ASIC2/3) in mouse sensory neurons in the dorsal root ganglion (DRG) that innervate the heart [28]. In contrast, we found that ASIC1/2/3 heteromers predominate in skeletal muscle sensory neurons [29]. Several studies investigating the role of ASIC2 in the periphery suggest that it might play a role in mechanosensation [30], [31], similar to the function of the related degenerin (DEG) channels in *Caenorhabditis elegans* [32], although the role of ASICs as mechano-transducers is uncertain [33], [34], [35]. In the CNS, ASIC2 subunits primarily form heteromultimers with ASIC1a. Electrophysiological studies of hippocampal, cortical, and spinal cord neurons identified acid-evoked currents that are the result of a combination of ASIC1a homomers, ASIC1a/2a heteromers, and ASIC1a/2b heteromers [5], [7], [36], [37]. Compared to ASIC1a homomers, ASIC1a/2a heteromers are slightly less pH sensitive and have faster kinetics of desensitization [3], [4]. When ASIC2b heteromultimerizes with ASIC1a in CNS neurons, ASIC2b imparts altered pharmacology and slight shifts in ion selectivity [37].

Despite the relatively subtle effects of ASIC2 subunits on the biophysical properties, recent studies demonstrate that ASIC2 is important for normal brain function. Genetic deletion of *ASIC2* generates neurological behavior deficits that mimic those seen in *ASIC1a −/−* mice [38]. Moreover, human genetic linkage studies have associated the *ASIC2* locus with autism, panic disorder, and lithium responsiveness in bipolar disorder [39], [40], [41]. Recent work has begun to elucidate the underlying mechanisms. ASIC2 facilitates ASIC channel localization to synapses on neuronal dendritic spines – sites of excitatory synapses. This effect, in part, is dependent upon ASIC2 interaction with the synaptic scaffolding protein, postsynaptic density-95 (PSD-95) [42]. Here, we explored the role of ASIC2 subunits on the expression of ASIC channels at the cell surface, and tested if PSD-95 can alter the functional expression of ASIC2 containing channels in both heterologous cells and hippocampal neurons.

## Materials and Methods

### Heterologous Expression of cDNA in CHO Cells

Mouse ASIC1a, mouse ASIC2a, and rat PSD-95 in pMT3 vectors were cloned as previously described [42], [43]. Human ASIC2b was kindly provided by Dr. Michael Welsh. HA-ASIC1a, HA-ASIC2a, and ASIC2a_GAA_ (replacement of the C-terminal 3 aa with Gly-Ala-Ala) were described earlier [42], [44]. DsRed (Express-C1) was purchased from Clontech, and green fluorescent protein (GFP; pGreen Lantern) was from Life Technologies. Chinese Hamster Ovarian (CHO) cells (ATCC, Manassas, Virginia) were cultured at 37°C, 5% CO_2_ in F12 nutrient medium (Gibco, Carlsbad, CA) supplemented with 10% fetal bovine serum and 1% penicillin/streptomycin. For electrophysiological studies, cells plated in 35 mm dishes at ∼10% confluence were transfected with ASIC cDNAs (0.18 μg/1.5 ml), PSD-95 or DsRed as control (1.82 μg/1.5 ml), and GFP (0.33 μg/1.5 ml) to facilitate detection of expressing cells by epifluorescence using lipid transfection reagent TransFast (Promega, Madison, WI) according to manufacturer’s recommendations. Coexpressed ASIC cDNAs were transfected at equal concentration for a total of 1.82 μg/1.5 ml. Cells were studied 2 days after transfection. Cells used for biochemistry were transfected with 7.5 μg of HA-ASIC1a or HA-ASIC2a and 7.5 μg of ASIC2a, ASIC2b constructs, or DsRed cDNA using Lipofectamine 2000 (Gibco, Carlsbad, CA) in 100 mm dishes. For experiments testing the effect of PSD-95 we transfected 7.5 μg of HA-ASIC2a or HA-ASIC1a and ASIC2a (total 7.5 μg), and 7.5 μg PSD-95 or dsRed cDNA. Our transfection protocol for electrophysiological studies has low efficiency, but transfected cells are easy to patch clamp and have large currents, whereas our transfection protocol for biochemistry is highly efficient to generate a large quantity of protein.

### Antibodies

The following antibodies were used: anti-HA-Peroxidase, High Affinity antibody (clone 3F10) (Roche Applied Biosciences, Indianapolis, IN), and anti-PSD-95 (6G6) (Santa Cruz Biotechnology, Santa Cruz, CA).

### Surface Biotinylation and NeutrAvidin Pulldown

48 hours after transfection, cells were incubated at 4°C for 5 minutes, followed by three washes at 4°C with PBS+ (1 mM PBS, pH 7.4; 1 mM MgCl_2_; 1 mM CaCl_2_), then incubated with 0.5 mg/ml sulfo-*N*-hydroxysuccinimide-biotin (Thermo Scientific Pierce, Rockford, IL) at 4°C for 30 minutes. Unbound biotin was quenched with 100 mM glycine in PBS+for 20 minutes at 4°C. The cells were lysed at 4°C for 20 min using 1% Nonidet P-40/63 mM EDTA/58.3 mM Tris-HCl pH 8/9.6 mM sodium deoxycholate, plus protease inhibitors (Sigma). The supernatant was pre-cleared with 50 μl of washed Protein A-Sepharose beads (Thermo Scientific Pierce, Rockford, IL) and then centrifuged at 16,100×*g* for 10 minutes at 4°C to remove insoluble material. 800 μg of lysate was tumbled with 30 μl NeutrAvidin-agarose beads (Thermo Scientific Pierce, Rockford, IL) for 24 hours at 4°C. After three washes, biotinylated proteins were eluted using SDS sample buffer (4% SDS; 100 mM DTT; 20% glycerol; 100 mM Tris·HCl, pH 6.8), separated by SDS/PAGE and then immunoblotted.

### Immunoprecipitation

Cells were washed at 4°C three times with PBS+ (1 mM PBS, pH 7.4; 1 mM MgCl_2_; 1 mM CaCl_2_), and then incubated for 20 min on a rocker at 4°C in 1 ml of lysis buffer [1% TX-100/50 mM Tris-HCl, pH 7.5/150 mM NaCl/1 Complete Mini protease inhibitor tablet/25–50 ml (Roche Applied Biosciences, Indianapolis, IN)]. Following centrifugation (16,100×*g*, 4°C, 10 min), the supernatant was pre-cleared with 50 μl of washed Protein A-Sepharose beads. Samples were tumbled with 1 μg of PSD95 antibody with 50 μl of Protein A-Sepharose beads. The immunoprecipitates were collected by centrifugation, washed three times with lysis buffer, and then resolved by SDS-PAGE.

### Immunoblotting

Samples were separated by SDS-PAGE on a 7.5% (w/v) gel and transferred to a Protran nitrocellulose membrane (Whatman, Dassel, Germany). The membrane was blocked in 5% bovine serum albumin (BSA) in TBS-Tween (0.05% (w/v) Tween-20 in 10 mM Tris, 100 mM NaCl, pH 7.5), and then incubated with the primary antibody (anti-HA-HRP, 1∶750) in 1% BSA/TBS-Tween. Membranes were developed using the Amersham ECL Plus Western Blotting Detection System (GE Healthcare, Buckinghamshire, UK). Bands were quantified with ImageJ.

### Electrophysiology

Whole-cell patch-clamp recordings (at −70 mV) from CHO cells and mouse hippocampal neurons were performed at room temperature with an Axopatch 200B amplifier (Axon Instruments, Foster City, CA) and were acquired and analyzed with PULSE/PULSEFIT 8.70 (HEKA Electronics, Lambrecht, Germany) and IGOR Pro 6.01 (WaveMetrics, Lake Oswego, OR) software or pCLAMP 8.2 (Axon Instruments). Recordings were filtered at 5 kHz and sampled at 2 or 0.2 kHz. Capacitive currents were compensated for and recorded for normalization of peak current amplitudes (reported as current densities). Micropipettes (2–4 MΩ) were filled with internal solution (mM): 100 KCl, 10 EGTA, 40 HEPES, 5 MgCl_2_, 2 Na_2_ATP, and 0.3 Na_3_GTP, pH 7.4 with KOH. External solution contained (mM): 120 NaCl, 5 KCl, 1 MgCl_2_, 2 CaCl_2_, 10 HEPES, 10 MES, pH adjusted with tetramethylammonium hydroxide, and osmolarity adjusted with tetramethylammonium chloride. Extracellular solutions were changed within 20 msec by using a computer-driven solenoid valve system [45]. Control solution (pH 8) flowed on cells for 30 s between acidic pH applications to allow for recovery from desensitization. Kinetics of desensitization was fit to single exponential equations and time constants (τ) reported. pH-dependent activation curves were fit to the Hill equation with IGOR Pro 6.01.

### Hippocampal Neuron Culture and Transfection

Primary hippocampal neuron cultures were prepared from postnatal day 0–2 mouse pups. Pups were placed on ice and then euthanized by rapid decapitation to minimize suffering. Hippocampi were rapidly dissected and digested for 2 min with 0.06% Trypsin-EDTA in Hank’s balanced salt solution without Mg^2+^ or Ca^2+^ (HBSS−/−; Gibco) plus glucose (6 mg/ml). Hippocampi were then washed with 10 ml followed by trituration in 2 ml HBSS−/− plus glucose. Following a 1-minute incubation, the upper portion of the suspension was transferred to a new tube (leaving behind ∼500 μl debris) and cells were pelleted by centrifugation (1000 rpm, 1 min). The pellet was then resuspended with plating medium [Neurobasal+B27+ Glutamax (Gibco) +5% horse serum], plated onto poly-lysine and laminin coated coverslips at a density of 4–6×10^5^ cells/35 mm dish, and maintained in a 37°C humidified 5% CO_2_ incubator. Medium was changed 3–4 hr later to culture medium (Neurobasal+B27+ Glutamax) and then changed every 3–4 days. Calcium phosphate mediated transfection was performed 5–6 days after plating. Before transfection, conditioned medium was saved and cells were washed with sequentially with HBSS with Mg^2+^ or Ca^2+^ (HBSS+/+; Gibco) and fresh culture medium. For each 35 mm dish, 7.5 μg of total DNA was diluted to a final volume of 20 μl with sterile ddH_2_O and mixed with 42.5 μl of 250 mM CaCl_2_. The DNA/Ca^2+^ mix was added dropwise to an equal volume of 2×HBS (mM: 50 HEPES, 280 NaCl, 1.5 Na_2_HPO_4_, pH 7.0) and mixed by pipetting. Precipitates were allowed to form by incubating at room temperature for 20–30 min and then the suspension was added dropwise to dishes. 1–2 hr after transfection, cultures were washed once with HBSS+/+ and twice with culture medium. Conditioned medium was then added back to cultures. Neurons were returned to the incubator and electrophysiological studies were performed 1–2 days later.

### Ethics Statement

All animal procedures and care were followed in strict accordance with the Guide for the Care and Use of Laboratory Animals of the National Institutes of Health and approved by the Institutional Animal Care and Use Committee of the University of Iowa. For preparation of primary hippocampal neuron cultures, postnatal day 0–2 mouse pups were placed on ice and then euthanized by rapid decapitation to minimize suffering.

### Data Analysis

Data are means ± SEM. Statistical significance between two groups was assessed using paired or unpaired Student’s *t*-test.

## Results

### ASIC2a Increases ASIC1a Cell Surface Expression and Current Amplitude

**Figure 1:**
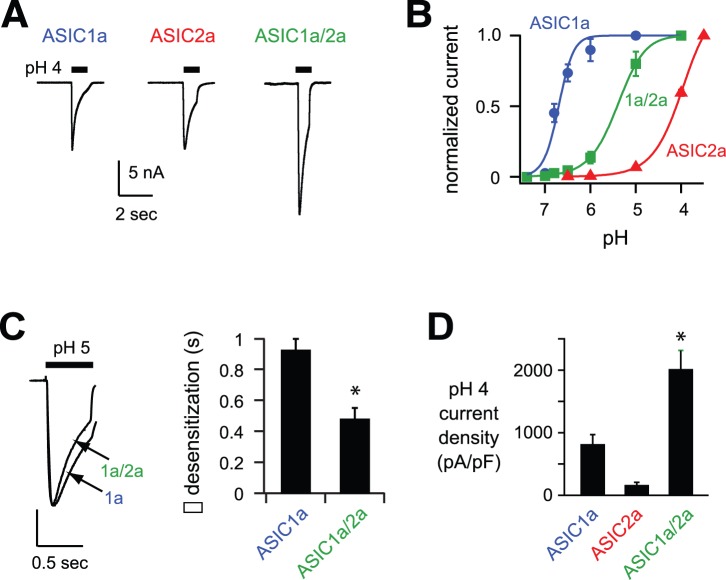
Coexpression of ASIC2a with ASIC1a generates increased currents. *A*, Representative acid-evoked currents generated by pH 4 application to CHO cells transfected with the indicated ASIC subunits. Coexpressed ASICs were expressed at half the normal amount to keep total ASIC cDNA constant. The bar above each current trace represents the time that the perfusing solution was rapidly changed from pH 7.4 to 4. *B*, pH-dose response curves for activation of cells expressing ASIC1a, ASIC2a, or coexpressing ASIC1a and ASIC2a. Data were acquired by stepping from pH 7.4 to the indicated test solution, and are normalized to the maximal currents (*n* ≥6). Lines are fits of the Hill equation. *C*, Superimposed pH 5-evoked currents from cells expressing ASIC1a or coexpressed ASIC1a and -2a (vertical scale bar: 3.66 nA ASIC1a and 5 nA coexpressed ASIC1a and -2a). To the right are the mean time constants (τ) of desensitization as measured from single exponential fits to the falling phase of currents evoked by pH 5 (*n* ≥8; *P<0.01). *D*, Mean current densities evoked by pH 4 in cells expressing the indicated ASIC subunits (*n* ≥6; *P<0.01 compared to ASIC1a expressed alone).

**Figure 2:**
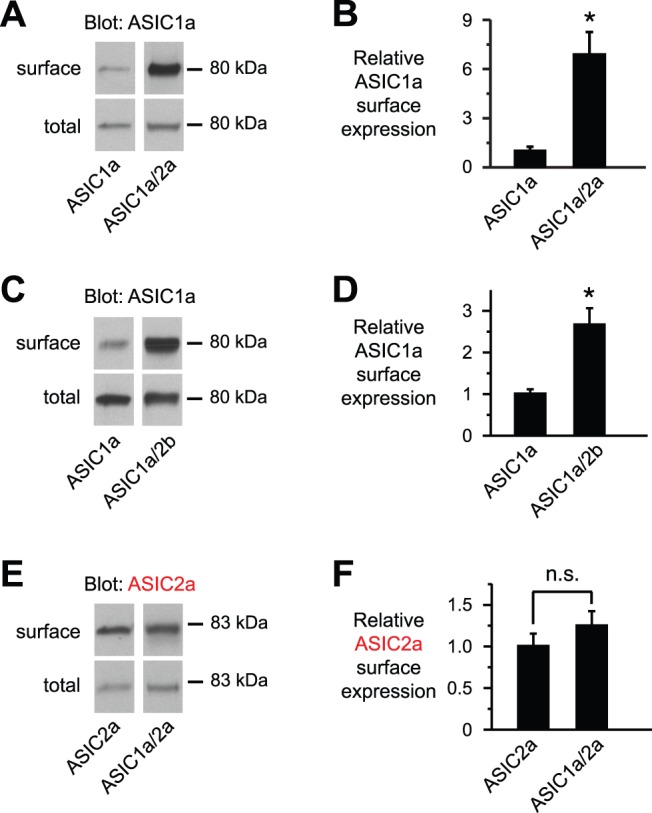
ASIC2 subunits increase ASIC1a cell surface expression. *A*, *C*, and *E*, Immunoblots (*IB*) (anti-HA) of biotinylated (*surface*) and total ASIC1a or -2a in CHO cells transfected with the indicated ASIC subunits (7.5 μg). The immunoblotted ASIC subunit was epitope-tagged with HA to allow detection. DsRed cDNA was added to keep total cDNA constant. Lanes for surface and total protein were from the same gel and film exposures respectively, but were rearranged for clarity. *B*, *D*, and *F*, Indicated ASIC surface expression is quantitated and normalized to the total amount of ASIC in the cell lysate for data from *A*, *C*, and *E*, respectively (*n* ≥5; *P<0.05; n.s. indicates no significant difference).

**Figure 3:**
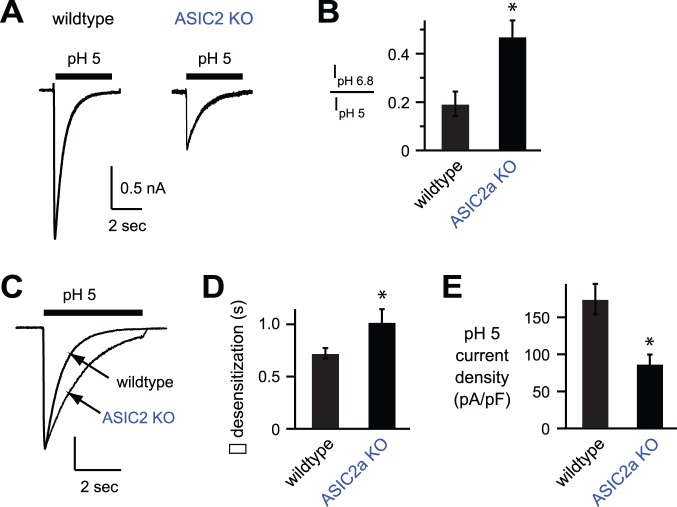
*ASIC2−/−* hippocampal neurons have reduced acid-evoked currents. *A*, Representative pH 5-evoked currents from wildtype and *ASIC2−/−* cultured mouse hippocampal neurons. *B*, The ratio of pH 6.8-evoked to pH 5-evoked current amplitudes as a measure of pH sensitivity (*n* ≥9; *P<0.01). *C*, Superimposed pH 5-evoked currents from hippocampal neurons of the indicated genotypes (vertical scale bar: 1 nA wildtype and 0.58 nA *ASIC2−/−*). *D*, Mean time constants (τ) of desensitization (*n* ≥11; *P<0.05). *E*, Mean current amplitude density evoked by pH 5 (*n* ≥11; *P<0.01).

We tested the functional effects of ASIC2a on ASIC currents when co-expressed with ASIC1a. For comparison, we also studied ASIC1a and ASIC2a homomers. When exposed to an acidic pH solution, CHO cells expressing either ASIC1a or ASIC2a subunits generated rapidly activating and desensitizing currents (Fig. 1A). However the properties of these currents differed markedly. As per previous reports, ASIC1a homomeric channels were activated by relatively modest pH changes (pH_50_ = 6.7), whereas ASIC2a homomers required much lower pH values for activation (pH_50_ = 4.0) (Fig. 1B and [3]). Coexpression of ASIC1a and -2a generated currents with properties consistent with heteromeric channels. The pH sensitivity of activation for the heteromeric channel was between that of the homomeric channels (pH_50_ = 5.4; Fig. 1B). In addition, pH 5-evoked current from ASIC1a/2a heteromers desensitized at a faster rate than ASIC1a homomeric channels (Fig. 1C). Interestingly, acid-evoked currents recorded from CHO cells transfected with both ASIC1a and -2a were consistently larger than those from cells expressing ASIC1a alone, even though the total concentration of transfected ASIC cDNA was equal (Fig. 2A and D). Of note, the amplitude of ASIC2a homomeric pH 4-evoked current is not reflective of the total number of channels at the cell surface because even at pH 3.5, the dose-response curve for activation of ASIC2a homomers has not reached a plateau (see Fig. 1B). On the other hand, pH 4 maximally activates ASIC1a and ASIC1a/2a channels.

The increase in current amplitudes when ASIC2a is coexpressed with ASIC1a could be due to an increase in conductance and/or an increase in channel expression at the cell surface. To test the later, we labeled ASIC1a at the cell surface with sulfo-NHS-biotin to allow isolation with Neutravidin. Compared to ASIC1a expressed alone, ASIC1a surface levels were increased when it was coexpressed with ASIC2a (Fig. 2A). When normalized to the total amount of ASIC1a in the cell lysate, coexpression of ASIC2a significantly increased the relative ASIC1a surface/total protein expression (Fig. 2B). We suspect that the greater increase in surface expression (∼7-fold), compared to the ∼2.5-fold increase in current (Fig. 1D), reflects a difference in the transfection protocols (see methods). We also tested the effect of the ASIC2 splice variant, ASIC2b, on ASIC1a cell surface expression. Like ASIC2a, -2b also increased the relative amount of ASIC1a at the cell surface (Fig. 2C and D). Conversely, ASIC1a did not increase the cell surface expression of ASIC2a (Fig. 2E and F). These results suggest that ASIC2 subunits facilitate expression of ASIC1a at the cell surface, and increase the number of functional channels available for activation.

Next we tested the effect of ASIC2 subunits on ASIC currents in cultured hippocampal neurons, where most ASIC2 subunits heteromultimerize with ASIC1a subunits to form H^+^-gated channels [5], [37]. pH 5 applications generated ASIC-like currents in most hippocampal neurons, and this was also true for neurons from mice that had targeted deletion of the *ASIC2* gene (Fig. 3A). As previously reported [5], loss of *ASIC2* generated currents that had significantly increased pH sensitivity as measured by the ratio of pH 6.8-evoked current to pH 5-evoked current amplitudes (Fig. 3B), and significantly slower desensitization kinetics (Fig. 3C and D). The properties of acid-evoked currents in *ASIC2−/−* neurons are consistent with those of ASIC1a homomeric channels [3], [5]. Importantly, and in agreement with our data in heterologous cells, ASIC currents in *ASIC2−/−* neurons were of significantly smaller amplitude than those from wildtype neurons (Fig. 3A and E). In summary, these data suggest that heteromultimerization of ASIC2 subunits with ASIC1a facilitate channel expression at the cell surface and increases acid-evoked current amplitudes.

### PSD-95 Reduces ASIC2a Cell Surface Expression and ASIC2a Acid-evoked Currents

**Figure 4:**
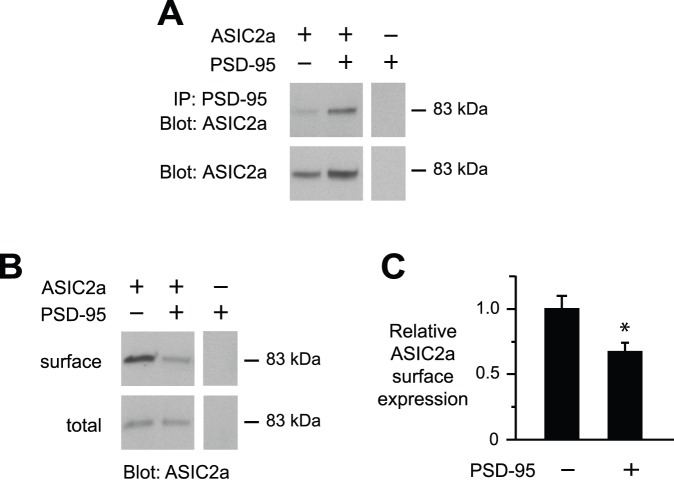
PSD-95 decreased ASIC2a cell surface expression. *A–C,* CHO cells were transfected with HA-ASIC2a and/or PSD-95, with DsRed added to keep total cDNA constant. *A*, Coimmunoprecipitation of ASIC2a with PSD-95. In the *top panels*, cell lysates were immunoprecipitated (*IP*) with anti-PSD-95 antibody, and then immunoblotted (*IB*) for ASIC2a (anti-HA). The *bottom panels* shows immunoblots of cell lysates. *B*, Immunoblot (anti-HA) of biotinylated (surface) and total ASIC2a. Lanes for surface and total protein in *A* and *B* were from the same gel and film exposure respectively, but were arranged for clarity. *C*, Quantification of relative ASIC2a surface expression with and without coexpressed PSD-95 (*n* = 9; *P<0.05).

ASIC2a can also interact with other proteins to modulate ASIC function and cell surface expression. We previously found that PSD-95 interacts specifically with ASIC2 in CNS neurons and targets ASIC channels to dendritic spines, the primary site of excitatory synapses [42]. Here we tested if PSD-95 alters the functional properties of ASIC2a. First we confirmed that ASIC2a interacts with PSD-95 when coexpressed in CHO cells. As shown in other cells [42], ASIC2a coimmunoprecipitated with PSD-95 (Fig. 4A). Next we tested if PSD-95 might alter ASIC2a cell surface expression. Figure 4B shows that PSD-95 decreased the amount of ASIC2a expressed at the cell surface without changing the total ASIC2a expressed in the cell (Fig. 4B), resulting in a significant decrease in relative ASIC2a cell surface/total protein expression (Fig. 4C).

**Figure 5:**
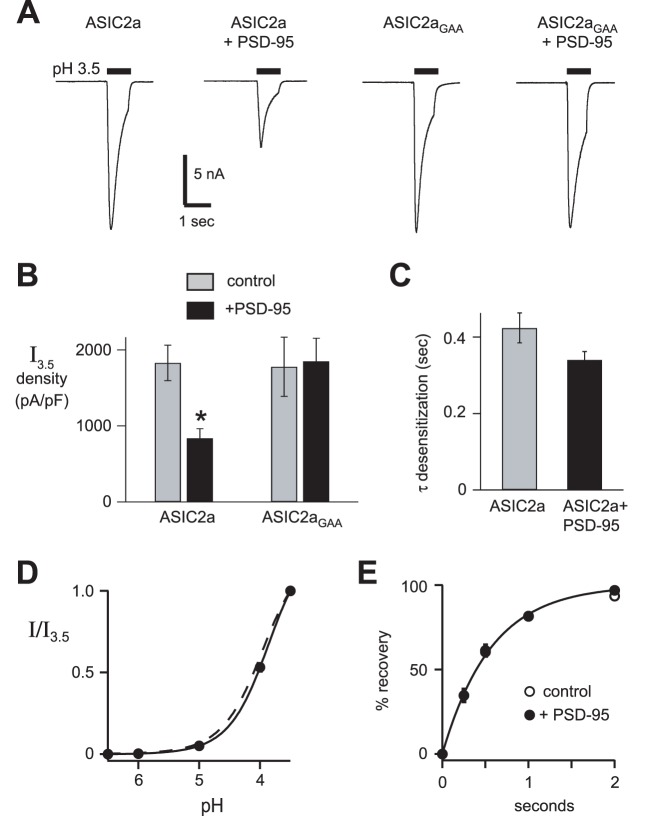
PSD-95 decreased ASIC2a acid-evoked current. *A*, Representative pH 3.5-evoked currents in CHO cells coexpressing wild-type ASIC2a or ASIC2a_GAA_ with either dsRed as a control or PSD-95 at 1∶10 cDNA ratios. *B*, Mean current density of currents evoked by pH 3.5 for above cells (*n* ≥12; *P<0.01 compared to ASIC2a without PSD-95). *C*, Mean time constants (τ) of desensitization of pH 3.5-evoked currents from ASIC2a in the presence and absence of PSD-95 (*n* ≥28). *D*, pH-dose response curves for activation of cells expressing ASIC2a and PSD-95 (pH_50_ = 3.9; *n* ≥12). Currents were normalized to those evoked by pH 3.5 for the dose-response data. Line is fit of Hill equation. Dashed line is fit of ASIC2a data from Fig. 1B. *E*, The mean time to recovery from desensitization. Data was collected by applying a prolonged 20 sec pulse of pH 4 to the cells to allow current to completely desensitize. pH 7.4 was then applied for the indicated times before applying a second pH 4 pulse. Recovery is the percentage of current evoked by the second pulse compared to the first. Line is fit of single exponential and is superimposed for the two groups (τ = 0.57 sec for each group; *n* ≥17).

We then tested if PSD-95 might modulate ASIC2a acid-evoked currents. As predicted from the surface expression data, coexpressing PSD-95 significantly reduced ASIC2a current amplitude (Fig. 5A and B). While PSD-95 reduced ASIC2a expression at the cell surface and decreased ASIC2a current amplitude, it did not significantly alter its biophysical properties including the rate of desensitization (Fig. 5C), pH sensitivity of activation (Fig. 5D), or recovery from desensitization (Fig. 5E). Thus, PSD-95 regulates ASIC2a by reducing its expression at the cell surface, and not by altering the biophysical properties of the channel. To test if this effect was dependent upon the PDZ-binding motif of ASIC2a, we mutated the three C-terminal amino acids of ASIC2a (Ile-Ala-Cys) to Gly-Ala-Ala (ASIC2a_GAA_). While ASIC2a_GAA_ generated acid-evoked currents with similar amplitudes as wildtype ASIC2a, mutation of the PDZ-binding motif abolished its capacity to be regulated by PSD-95 (Fig. 5A and B).

### PSD-95 Alters ASIC1a Cell Surface Expression and Current Amplitudes when Coexpressed with ASIC2a

**Figure 6:**
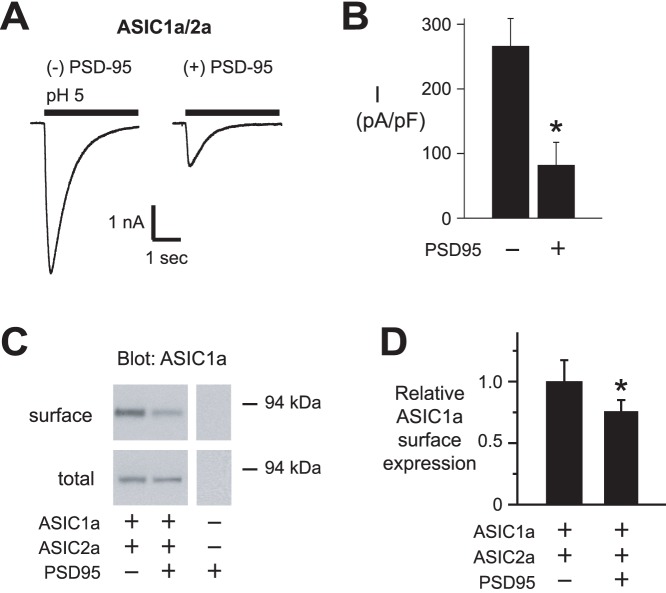
PSD-95 inhibits ASIC1a/2a heteromeric channels. *A* **,** Representative pH 5-evoked currents in CHO cells coexpressing ASIC1a and ASIC2a, with either dsRed as a control or PSD-95 at 1∶10 cDNA ratios. *B*, Mean current density of currents evoked by pH 5 for above groups of cells (*n* ≥7; *P<0.01 compared to without PSD-95). *C*, Immunoblot (anti-HA) of biotinylated (surface) and total ASIC1a when coexpressed in CHO cells with ASIC2a, with and without PSD-95. Lanes for surface and total protein in *A* were from the same gel and film exposure respectively, but were arranged for clarity. *D*, Quantification of mean relative surface to total ASIC1a (*n* ≥5, *P<0.05).

Previous work demonstrated that PSD-95 does not interact with ASIC1a and does not alter ASIC1a homomeric channel function [42], [46]. Since coexpression ASIC1a and ASIC2a predominantly results in heteromeric channels composed of both subunits (Fig. 1), we asked if PSD-95 might modulate ASIC1a if it was coexpressed with ASIC2 subunits. Figures 6A and B demonstrate that coexpression of PSD-95 with ASIC1a and -2a generated currents with smaller amplitudes compared to currents generated by ASICs expressed alone. Consistent with a reduction in current amplitudes, coexpression of PSD-95 along with ASIC2a, reduced the relative amount of ASIC1a at the cell surface (Fig. 6C and D).

### PSD-95 Decreases ASIC1a/2 Acid-evoked Currents in Hippocampal Neurons

**Figure 7:**
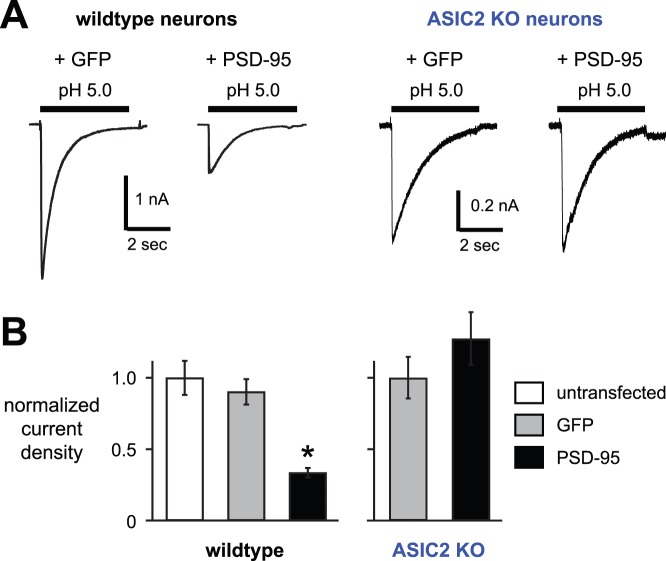
PSD-95 decreases acid-evoked currents in hippocampal neurons, and is dependent upon *ASIC2*. *A*, Representative acid-evoked currents from wildtype or *ASIC2−/−* cultured mouse hippocampal neurons transfected with either GFP alone (*GFP*), or GFP and PSD-95 (*PSD-95*). *B*, Mean current densities evoked by pH 5.0 in the indicated groups of hippocampal neurons (*n* ≥10; *P<0.01 compared to both wildtype untransfected and GFP groups).

Our data in heterologous cells demonstrated that ASIC2a, when it is a component of a heteromeric ASIC channel, imparts the capacity to be modulated by PSD-95. In CNS neurons, most ASIC2 subunits heteromultimerize with ASIC1a subunits to form H^+^-gated channels [5], [6]. To test the effects of PSD-95 on native channels, we overexpressed PSD-95 in cultured hippocampal neurons, and studied their acid-evoked currents. Figures 7A and B show that overexpression of PSD-95 significantly reduced the amplitude of pH 5.0-activated currents, whereas expression of GFP as a control had no effect. Although the currents were smaller, PSD-95 did not alter the pH sensitivity (as measured by the pH 6.8 to pH 5.0 current amplitude ratio; 0.185±0.045 for the GFP and 0.150±0.026 for PSD-95 groups). Also, the kinetics of desensitization of pH 5-evoked currents (τ = 0.72±0.05 s untransfected, 0.72±0.04 s GFP, 0.74±0.05 s PSD-95; *n* ≥12), and recovery from desensitization (% recovery at 1 s = 0.29±0.09 GFP, 0.22±0.04 PSD-95; *n* ≥7) were unchanged by PSD-95 overexpression. Based on our data to this point, we hypothesized that PSD-95 would have no effect in hippocampal neurons that lacked ASIC2 subunits. Indeed, overexpression of PSD-95 in *ASIC2−/−* neurons had no effect on acid-evoked currents (Fig. 7A and B). Thus, PSD-95 inhibition of ASIC current in hippocampal neurons was dependent on ASIC2.

## Discussion

Although *ASIC2* was the first ASIC to be cloned almost two decades ago [24], its function has remained rather elusive. ASIC2a homomeric channels require very low pH values for activation (pH_50_ = 4.0 in this study), which is probably below the range achieved *in vivo* during physiological and even pathological conditions. ASIC2b does not form homomeric acid-gated channels. Moreover, when ASIC2a heteromultimerizes with other ASIC subunits, it generally reduces channel pH sensitivity, and when ASIC2b heteromultimerizes with other subunits, the pH sensitivity of activation is unaltered. Thus, ASIC2 subunits themselves probably do not serve as pH sensors. And yet, recently published data showed that targeted deletion of *ASIC2* produced behavioral phenotypes that were remarkably similar to those seen in *ASIC1a −/−* mice [38]. Loss of either *ASIC1a* or *ASIC2* similarly diminished unconditioned fear responses to predatory odor or CO_2_, and both genotypes showed similarly reduced conditioned fear behavior. In addition, *ASIC1a* and *ASIC2* null mice demonstrated less depressive-like activity in a forced swim assay. On the whole, deletion of either *ASIC1a* or *ASIC2* generated equal and maximal behavioral deficits, as mice that had combined loss of *ASIC1a* and *ASIC2* did not display a greater phenotype than deletion of the individual genes.

These results raise the question: how does loss of *ASIC2* generate behavioral phenotypes that mimic the loss of *ASIC1a*, when ASIC2 subunits contribute relatively little to the pH sensitivity of CNS neurons? Our results here lend insight. In heterologous cells, expression of ASIC2 subunits increased the surface expression of ASIC1a and increased acid-evoked current amplitude. In cultured hippocampal neurons, disruption of *ASIC2* reduced current amplitude. Thus, when ASIC2 subunits heteromultimerize with ASIC1a, more channel protein is expressed at the cell surface, which can increase neuronal responses when exposed to local acidosis. However, it should be noted that a previous study did not show a difference in acid-evoked current amplitudes recorded from *ASIC2−/−* hippocampal neurons when compared to wildtype [5]. The reason for this difference is unclear. Consistent with our data here, ASIC2 facilitated the localization of ASICs to dendritic spines in brain slices of the hippocampus, and enriched ASIC expression in brain synaptosomal membrane fractions. Moreover, deletion of *ASIC2* reduced acid-evoked intracellular Ca^2+^ elevations in dendritic spines [42]. These findings suggest an important role of ASIC2 subunits in the trafficking and localization of ASIC channels to synapses and perhaps extra-synaptic membrane sites where they can be activated by local changes in pH.

While our data support a role for ASIC2 in the subcellular localization of ASIC channels, changes in the biophysical channel properties conferred by ASIC2 subunits might also be important. For example, the faster kinetics of desensitization, and in particular the faster recovery from desensitization of ASIC1a/2a heteromers compared to ASIC1a homomers, might be important for repeated activation of ASICs under fluctuating pH conditions. The importance of desensitization kinetics for channel function is illustrated by the association of an inheritable form of epilepsy with mutant nicotinic acetylcholine receptors that display faster desensitization and slower recovery from desensitization [47].

How do ASIC2 subunits facilitate subcellular localization of ASIC channels? Our studies demonstrating a functional interaction between ASIC2 and the neuronal scaffolding protein PSD-95 provide clues. ASIC subunits differ most in their intracellular N- and C-termini. Within these cytoplasmic regions, several subunit specific protein interaction motifs have been identified that regulate ASIC trafficking and function. The C-terminal residues of ASIC1, -2, and -3 each contain distinctive PDZ-binding motifs that allow for interaction with different cytosolic PDZ domain-containing proteins. For example, ASIC1 and -2 subunits, but not ASIC3, interact with PICK1 allowing for modulation by PKC [48], [49], [50]. On the other hand, ASIC2 and -3, and not ASIC1 subunits, interact with PSD-95 to effect subcellular localization [42], [46]. Other PDZ proteins that bind to the PDZ-binding motifs of various ASICs and modulate their function include CIPP, NHERF, LIN-7b, MAGI-1b, and PIST [46], [51], [52]. Other cytoplasmic regulatory domains within specific ASICs have also been described. Phosphorylation of Ser-25 within the N terminus of ASIC1a, and probably ASIC2a, by a PI3-kinase/Akt pathway regulates channel exocytosis [53]. A juxtamembrane region within the C terminus of ASIC1 is required for clathrin-dependent endocytosis [54]. Recent reviews describe additional examples [55], [56]. We found that coexpressing ASIC2 subunits in CHO cells increased surface expression of ASIC protein and increased current amplitudes, and perhaps the underlying mechanism involved ASIC2 interaction with endogenous cytosolic proteins. We speculate that heteromultimerization of different ASIC subunits increases the opportunity for interaction with multiple different cytosolic proteins.

Alternatively, heteromultimerization might be preferred in the biosynthesis, assembly, and/or trafficking of ASIC channels. Such is the case for the related epithelial Na^+^ channel (ENaC), a channel that transports Na^+^ to maintain normal salt and water homeostasis [57]. ENaC is composed of α, β, and γ subunits, and expression of all three subunits is necessary for maximal cell surface expression and function [58]. Studies of native acid-evoked currents in various populations of neurons support the idea that ASICs are also preferentially assembled as heteromultimers [3], [5], [7], [28], [29], [36], [37]. In the future it would be interesting to investigate if ASIC heteromultimerization alters the efficiency of channel processing within biosynthetic pathways.

Similar to its effect on ASIC3 [46], [59], we found that PSD-95 reduced cell surface expression and acid-evoked current amplitude in channels containing ASIC2. Consistent with previous work [42], mutation of the C-terminal PDZ-binding domain of ASIC2a abolished the capacity of PSD-95 to modulate ASIC2a. However, the mechanism by which PSD-95 decreases ASIC cell surface levels remains largely unknown. We previously demonstrated that PSD-95 modulation of ASIC3 was dependent upon their interaction within lipid rafts – membrane microdomains rich in cholesterol and sphingolipids that organize receptor/signaling complexes [59]. Moreover, our data suggested that most ASIC3 at the cell surface, independent of its interaction with PSD-95, is expressed within lipid raft membrane fractions [59]. Perhaps ASIC2 is similarly regulated by PSD-95 within lipid raft membrane microdomains.

Consistent with our data in heterologous cells, PSD-95 decreased acid-evoked current amplitude in hippocampal neurons in an ASIC2 dependent manner. Previous data showed that PSD-95 targeted ASIC2 to post-synaptic sites on dendritic spines [42]. We speculate that these results represent PSD-95 targeting ASIC expression to different locations with the neuron. PSD-95 organizes ion channels with their associated signaling partners at post-synaptic membranes, thus regulating the strength of synaptic activity [60]. ASICs are preferentially distributed in neuronal cell bodies, along dendritic shafts, and at dendritic spines [6], [61]. Since whole-cell patch-clamp of cultured neurons measures current through channels primarily expressed at the cell body, it is interesting to speculate that overexpression of PSD-95 signals the translocation of ASIC channels out of the cell body to dendritic spines.

In summary, accumulating evidence suggests that ASIC2 subunits are integral components of ASIC channels and contribute to normal brain function. Genetic deletion of *ASIC2* in mice generates neurological behavior deficits that mirror those seen in mice lacking *ASIC1a*, which is the major pH-sensitive ion channel subunit in central neurons [38]. Moreover, recent human genetic linkage studies have associated the *ASIC2* locus with multiple neurological disorders [39], [40], [41]. Heteromultimerization with ASIC2 subunits not only affects the biophysical properties of acid-evoked currents, but perhaps more importantly, our data here together with other recent studies suggest an important role for ASIC2 subunits in subcellular localization of the channels. Further studies of ASIC biosynthesis, assembly and trafficking will lend mechanistic insight into the role that ASICs play within the nervous system.
